# Recurrent Vulvovaginal Candidiasis: Assessing the relationship between feminine/vaginal washes and other factors among Ghanaian women

**DOI:** 10.1186/s12889-024-17668-x

**Published:** 2024-01-05

**Authors:** Emmanuel Otoo-Annan, Vivian Efua Senoo-Dogbey

**Affiliations:** 1 Ghana Institute of Management and Public Administration (GIMPA) School of Public Service, Accra, Ghana; 2 Department of Public Health, School of Nursing and Midwifery, University of Ghana, P. O. Box LG 25, Legon, Accra, Ghana

**Keywords:** Feminine/vaginal wash, Prevalence, Vulvovaginal candidiasis, Recurrent, Women

## Abstract

**Introduction:**

Vulvovaginal Candidiasis (VVC) is a public health problem, with approximately 30–50% of women affected at least once during their lifetime. Recurrent Vulvovaginal Candidiasis (RVVC) is diagnosed following three or four repeated episodes of VVC in a calendar year. This condition poses health concerns with significant impacts on the quality of life of women. This cross-sectional study estimated the prevalence of RVVC and assessed the relationship between feminine/vaginal washes and other factors on RVVC among Ghanaian women in the Sekondi/Takoradi Metropolis.

**Methodology:**

A cross-sectional study was employed to gather data from 304 women. Data were collected using a pretested questionnaire. Bivariate and multivariate analyses, including chi-square/Fisher’s exact test and logistic regression, were performed using Jamovi (R Core Team 2021) software. Proportions were calculated, and odds ratios and their corresponding 95% confidence intervals were computed with the level of significance set at 0.05.

**Results:**

The prevalence of RVVC was estimated as 48.4% (95% CI 42.6%, 54.1%). Feminine Vaginal wash use (aOR = 3.86; 95% CI = 2.18, 6.84); age 36–45 years (aOR = 0.36; 95% CI = 0.17, 0.76) marital status (aOR = 2.37; 95% CI = 1.17, 4.79) and Sexual activity (aOR:0.43: 95%CI = 0.21, 0.88) were significantly associated with RVVC with *p* < 0.005.

**Conclusion:**

RVVC is prevalent among women in the Sekondi/Takoradi Metropolis of Ghana. Feminine/Vaginal washes could be cautiously linked to the development of RVVC.

## Introduction

The Centers for Disease Prevention and Control (CDC) defines a woman having three or more episodes of symptomatic episodes of Vulvo Vaginal Candidiasis (VVC) in less than twelve months as having Recurrent Vulvovaginal Candidiasis (RVVC) [1, 2]. RVVC has a significant economic burden, as most conventional antimycotics are sparingly active when treating these infections [3]. Researchers have observed that close to 10 to 20% of all women with RVVC are infected by *Candida glabrata* and other *Candida albicans* species [4]. The pathogenesis of RVVC is yet to be fully understood since most women do not have underlying conditions or apparent predisposing factors that could increase their risk of repeated episodes [5].

RVVC is a complex multifactorial disorder resulting from a change in the vaginal flora composition, host predisposing, genetic, and environmental factors as well as the kind of strain of *Candida* involved [6]. RVVC places a significant health burden on the individual, such as reduced quality of life and a financial burden on the health system. RRVC also puts a significant psychological strain on infected women [7]. There are reports of depression, stress and anxiety being significantly higher in women with RVVC than in those without [3, 8]. RVVC also has consequences for women of reproductive age. For example, during pregnancy, untreated RVVC may result in systemic infections in neonates, preterm births, low birth weight and related mortalities [9].

In Ghana, the problem of RVVC could even be more significant than the risk among women in advanced countries. Ghana’s health systems and facilities are less robust and less advanced than those in developed countries. The laboratory diagnosis, including vaginal swabs, culture and sensitivity testing, and the lack or difficulty in accessing health care services and specialist care in Ghana could affect the treatment outcomes and collation of relevant statistics for developing the right interventions [10].

Worldwide prevalence and epidemiological data are rare and inaccurate because they are mostly carried out from self-reports and local general practitioner diagnoses [6]. The situation is not different in Ghana, with just a handful of studies in the area of RVVC [11–14]. Information on the burden of RVVC and the relationship between vaginal wash use and RVVC is rare, especially in the Sekondi-Takoradi municipality.

The use and marketing of various feminine/vaginal washes, tightening creams and douches on the Ghanaian market has recently skyrocketed. These products are primarily marketed as good hygiene products and are used with the perceived effect of giving the vagina a pleasant smell, cleaning and tightening the vagina, among many others. The use of famine/vaginal washes is a common practice among women globally even though researchers have demonstrated that such a practice offers women no benefits but rather harms [15].

This study, therefore, sought to estimate the prevalence of RVVC and understand the relationship between the use of feminine/vaginal wash products and other factors and RVVC.

## Materials and methods

### Study design

This study employed a cross-sectional hospital-based analytical approach. This took a snapshot of the study population without influencing any of the variables in the study. This observational study measured both the exposure and the effect simultaneously.

### Study area

This study was conducted in the Sekondi/Takoradi Metropolis. The twin city is called an industrial and commercial center, with a population of 445,205 people. It is the capital of Sekondi–Takoradi Metropolitan Assembly and the Western Region of Ghana. Five health facilities were selected from the study area. The study sites included Effia Nkwanta Regional Hospital, Essikado Hospital, Takoradi Hospital, Ghana Ports and Harbors Authority Hospital, and UQ Specialist Medical Services. Apart from UQ Specialist Medical Services, which is a private institution, and the Ports and Harbours Hospital, which is a Quasi-Government hospital, all the other three facilities are government-owned and operate under the Ghana Health Service. The deliberate selection of the study sites made it possible for a good representation of a regional hospital, district hospital, quasi-government hospital and a private hospital.

### Study population

The target population was women in the Sekondi/Takoradi metropolis. The study population included women who reside in the Sekondi/Takoradi metropolis who reported to any of the 5 study sites for treatment, antenatal or review on account of symptoms such as vaginal discharge, itching and irritation, change in colour of vaginal secretions, pain in micturition and dyspareunia. Women within the 15–49 years age bracket who had received an official diagnosis of vaginal candidiasis from a qualified doctor by requesting high vaginal swabs for microscopic examination and other confirmatory laboratory procedures participated in the study. Women with Human Immune Deficiency Virus (HIV) and those with compromised immune systems (e.g. those on chemotherapy) were excluded.

### Sample size

This study used a confidence interval of 95% with a corresponding z-value of 1.96. The margin of error (d) = 0.05 and proportion (P) = 23.5% to compute a minimum sample size. The proportion was taken from a related study in Abidjan in 2019 [16]. Using the formula for estimating proportions in cross-sectional studies [17] and 10% allocation for nonresponse and missing data, a minimum sample size of 304 samples was deemed appropriate for this study.

### Participant recruitment and sampling

The study sites were deliberately selected for us to have representation of regional, district, quasi-government, and private facilities to allow for heterogeneity in the study samples. The total sample size for the study was proportionally allocated to the five study sites using their caseloads or O.P.D. per capita. The facility- or study site-specific sample size was obtained by proportional allocation of sample procedures, which was achieved by dividing the caseloads of each study site in the past month by the total (summation of) cases seen in all five facilities during that same period and multiplying by 304, which is the minimum sample size estimated for this study. Data collection took place two times a week. On such days, women who reported and were diagnosed with VVC were approached in the consulting rooms. Simple random sampling (lottery method) was utilized to recruit the participants once the person accepted and consented to be included in the study. The women were given a bag with folded pieces of paper with indications of *YES* and *NO* in equal numbers. The women dipped their hands in the bag, and those who chose *YES* were recruited into the study. We recruited the study participants serially at each study site until the sample size allocated for each study site/facility was attained.

### Data collection instrument

This study used a questionnaire to collect data from the participants. The questionnaire had closed-ended questions. The questionnaire had four sections. The first part focused on demographic variables (age, marital status, educational attainment, etc.). The second part looked at the medical history of the participants (antibiotic and steroid use, diabetes, etc.). The third part examined the occurrence of repeated episodes of vulvovaginitis among women. The fourth part evaluated the use of feminine/vaginal washes. The instrument was pretested on 10 participants in a facility other than those selected for the study. The responses generated were analysed to ascertain the respondents’ understanding of the survey questions. The pretest provided valuable information that was used to check for the validity of the instrument. Content validity was ensured such that all the questions in the data collection instrument were relevant, comprehensive, and appropriate for measuring the intended constructs. The pretesting also helped to ensure that the questions were comprehensible and logical to the respondents. The supervisor of the project undertook an expert review of the instrument, and suggestions were implemented. The data collectors went through an orientation process to familiarize themselves with the instrument before the actual survey.

### Data collection procedure

Participants were approached as they reported to the medical and gynecological units of the study sites. The aims of the study were explained to them, and their consent for participation was sought. The inclusion and exclusion criteria were applied at each instance, and participants who were eligible and demonstrated their interest in participating in the study were recruited randomly using the lottery method. They were then taken to consulting rooms at the general O.P. D or focused antenatal cubicles (for those who were recruited through the gynecological units) where face-to-face interviews were performed, and responses were entered into the questionnaires. The interviews were conducted by the principal investigator and his research assistants, with the entire interaction lasting for 30 min on average.

### Study variables and data analysis procedures

#### Study variables

RVVC was classified as having three episodes of VVC in a calendar year as per CDC STI treatment guidelines [2]. Feminine/vaginal wash use was classified as utilizing any preparation other than water for different purposes including cleaning, freshening up and giving a pleasant smell and tightening the vaginal canal with a frequency of 2 times a week in the last 12 months.

#### Statistical analysis and procedures

Data gathered through questionnaires were input into Jamovi (R Core Team 2021) for analysis. Frequency distributions, percentages, means, and proportions were computed for the study variables as needed. Bivariate and multivariate analyses were performed and Odds Ratios (OR) with their corresponding Confidence Intervals (CI) were computed to estimate associations between RCVV and the independent variables.

### Ethical considerations

Ethical clearance was obtained from the Ghana Institute of Management and Public Administration (GIMPA). Official letters from the health administration of the study sites were obtained granting permission for data collection to be undertaken. The participants were informed about the study’s objectives, and valid and written informed consent was obtained directly from participants and legal guardians of those below 18 years before the data collection could begin. The respondents had the right to refuse, withdraw, or terminate at any point without giving any reason. The information provided by the respondents was treated with the highest degree of confidentiality.

## Results

### Sociodemographic characteristics of the participants

Table 1 presents the participants’ sociodemographic characteristics. Most of the participants fell within the 26 to 35 age range (45.4%). Regarding marital status, a significant portion of participants were single (62.2%), while 33.9% were married. The educational level distribution indicated that 42.1% had attained tertiary education, and 43.8% had completed high school. Close to 70% of the participants were employed. The income level distribution indicates that the largest group (24.7%) falls within the income range of between 2000 and 3000, followed by between 1000 and 2000 (23.7%).

**Table 1:** Summary of demographic data of respondents

Variable	N (304)	Percentage (%)
**Age Group**		
15 to 25	99	32.6
26 to 35	138	45.4
36 to 45	67	22
**Marital Status**		
Single	189	62.2
Married	103	33.9
Divorced	7	2.3
Widowed	5	1.6
**Educational Level**		
Postgraduate	32	10.5
Tertiary	128	42.1
High School Level	133	43.8
Pre-High School	11	3.6
**Employment Status**		
Employed	211	69.4
Unemployed	93	30.6
**Income Level**		
Less than 1000	41	13.5
Between 1000 and 2000	72	23.7
Between 2000 and 3000	75	24.7
Above 3000	54	17.8
None	62	20.4

### Medical history of participants

As shown in Table 2, the majority (92.4%) did not have chronic illnesses. Only a tiny proportion (4.3%) reported having a form of chronic illness. Only 11.8% reported that they had been pregnant in the last year. Very few participants (0.7%) underwent hysterectomy. An overwhelming majority, 91.4%, were not on any medication. Similarly, antibiotics and oral steroid use were negative for 96.4% and 99.7% of the participants, respectively. Only 15 (4.9%) were on oral contraceptives. The participants unanimously reported not being cigarette smokers (100%). Regarding alcohol consumption, the majority (93.4%) indicated that they did not consume alcohol. Concerning sexual activity, a substantial proportion reported engaging in protected sexual activities (38.2%), while 33.6% reported engaging in unprotected sexual practices.

**Table 2:** Medical History

Variable	N (304)	Percentage (%)
Chronic illness*		
Negative	281	92.4
Positive	13	4.3
Do not know	10	3.3
**Pregnancy in the last year**		
Negative	268	88.2
Positive	36	11.8
**Hysterectomy**		
Negative	302	99.3
Positive	2	0.7
**Currently on Medication**		
Negative	278	91.4
Positive	26	8.6
**Antibiotic use**		
Negative	293	96.4
Positive	11	3.6
**Oral Steroid use**		
Negative	303	99.7
Positive	1	0.3
**Oral Contraceptives use**		
Negative	289	95.1
Positive	15	4.9
**Cigarette Smoking**		
No history of smoking	304	100
History of smoking	0	0
**Alcohol Consumption**		
Does not consume	284	93.4
Consumes	20	6.6
**Sexual Activity**		
Unprotected sexual activity	102	33.6
Choose not to answer	86	28.3
Protected Sexual activity	116	38.2

*Chronic Disease Chronic diseases are defined broadly as conditions that last 1 year or more and require ongoing medical attention or limit activities of daily living or both (Hypertension, diabetes, cancers etc.) HIV Cases were excluded.

### Prevalence of recurrent vulvovaginal candidiasis and feminine/vaginal wash use

Table 3 presents the prevalence of RVVC and the usage of Feminine/Vaginal wash among the participants, along with corresponding 95% confidence intervals. In terms of RVVC, among the total participants, 147 were classified as having RVVC (individuals who had experienced VVC three or more times within the past twelve months) giving RVVC prevalence of 48.4% (CI: 42.60, 54.10%). The prevalence of feminine/vaginal wash use was 47.7% (CI: 42.0%, 53.5%).

**Table 3:** Prevalence of VVC, RVVC and Feminine/Vaginal wash use

					95% Confidence Interval	
Prevalence		Count	Total	Percentage	Lower	Upper
**Prevalence of RVVC** Recurrent VVC		147	304	48.4	42.6	54.1
No Recurrent VVC		157	304	51.6	45.9	57.4
**Prevalence of** **Feminine/vaginal wash use**						
Feminine wash use Positive		145	304	47.7	42.0	53.5
Feminine wash use Negative		159	304	52.3	46.5	58.0

### Factors Associated with RVVC

The results presented in Table 4 show that none of the medical history variables showed any statistically significant association with RVVC at the bivariate stage. However, in controlling for all the confounders identified in this study, feminine/vaginal wash use, marital status and age were seen to be significantly associated with the development of RVVC at the multivariate stage. The study revealed that women who used feminine was had almost 4 times higher odds of developing RVVC (aOR = **3.86 95%; CI = 2.18, 6.84)**. Women between 36 and 45 years had lower odds of developing RVVC (aOR = **0.36;** 95% **CI = 0.17, 0.76; *p* = 0.007).** Married women had higher odds of developing RVVC compared to their unmarried counterparts (aOR = **2.37; 95% CI = 1.17, 4.79; *p* = 0.004)**. Sexually active women also demonstrated lower odds of RVVC (aOR=:0.43 95%CI = 0.21, 0.88; *p* = 0.021) compared to the sexually inactive ones (Table 4*).*

**Table 4:** Factors associated with RVVC among respondents

	RVVC		Crude Estimates				Adjusted Estimates			
				95% CI				95% CI		
Predictor	Yes	No	Odds ratio	Lower	Upper	*p*	Odds ratio	Lower	Upper	*p*
**Feminine wash**:										
No**	55	104	1.00				1.00			
Yes	92	53	3.28	2.05	5.25	< 0.001	3.86	2.18	6.84	**< 0.001**
**Age Group**:										
26 to 35**	58	80	1.00				1.00			
15 to 25	47	52	1.43	0.82	2.52	0.211	0.70	0.37	1.34	0.283
36 to 45	42	25	2.51	1.32	4.79	0.005	0.36	0.17	0.76	**0.007**
**Marital Status**:										
Single**	94	95	1.00				1.00			
Married	42	61	0.6	0.35	1.02	0.058	2.37	1.17	4.79	**0.004**
Divorced	7	0	1.24	0.10	2.12	0.986	0.58	0.66	0.90	0.986
Widowed	4	1	5.73	0.61	54.04	0.127	0.15	0.01	1.79	0.133
**Sexual Activity**:										
No**	*51*	65	1.00				1.00			
Yes	56	46	1.44	0.81	2.56	0.212	0.43	0.21	0.88	**0.021**
Choose not to answer	40	46	1.26	0.70	2.29	0.442	0.60	0.30	1.20	0.147

** reference category

## Discussion

Recurrent Vulvovaginal Candidiasis (RVVC) is a condition that is difficult to treat, and complete eradication is very difficult [18]. Antimycotics are used to suppress the symptoms when they appear. It is characterized by three or more Vulvovigina Candidiasis (VVC) episodes within a calendar year [2, 9].

There are inconsistencies in the case definition for RVVC. In the European region, and per the Infectious Diseases Society of America guidelines, women require four or more symptomatic episodes of VVC in a year for a diagnosis of RVVC to be made [20–22]. However, according to current CDC STI treatment guidelines, RVVC is diagnosed based on 3 or more repeated episodes of VVC [2]. We aligned this present study with the case definition of 3 or more episodes of VVC by the CDC and estimated the prevalence of RVVC to be 48.4% among patients who reported to the study sites and were clinically diagnosed with VVC. The prevalence estimated in this study is nearly four times greater than the prevalence reported among European and U.S. women, which was documented at 9.0% by Foxman (2013) et al. [23]. Likewise, our findings surpass the global estimate for RVVC of 7.0% (138 women annually) derived from a systematic review conducted by Denning et al. (2018), which encompassed 8 studies involving 17,365 patients from 11 countries [7]. Notably, the substantial variance in the findings may be attributable to the application of different case definitions in those studies, wherein RVVC is defined by four or more episodes of VVC within a year.

On the other hand, a more recent study in the United States by Yano et al. (2019) reported a high prevalence of RVVC at 34.0%. The present study bears a similarity with Yano et al.’s (2019) study in terms of the design [1]. The findings from these two related studies confirm the existing debate that the actual global prevalence of RVVC may be much higher than the 10.0% estimated by Denning et al., (2018) [7].

Our study found a statistically significant association between RVVC and the age of the participants such that women between age 35–45 years had lower odds of developing RVVC compared to their younger counterparts. This observation agrees with findings from a recent systematic review and meta-analysis that reported a lower prevalence of RVVC in individuals above the age of 34 + years [7]. Blonstein et al., (2017) in their review based on an internet panel survey involving 7345 women from seven countries reported a lower prevalence of RVVC in older women compared to the 19–25-year groups. The authors argued that the prevalence of RVVC was lower in older women because they were unable to remember the episodes of VVC they had in the past [24]. Inferring from this argument, the ability to recall the number of VVC episodes in the immediate past could influence the estimates in this present and many other studies.

Our study revealed a higher odds of RVVC among married women compared to their unmarried counterparts. This observation agrees with reports from an online survey involving 4548 women from the USA which identified married women as being at greater risk of VVC [25]. Several studies have reported sexual activity as a risk factor for RVVC in women [25] and perhaps this observation forms the basis of classifying RVVC as a sexually transmitted infection. Ironically, in this present study, sexually active women rather demonstrated lower odds of RVVC. This result aligns with researchers who believe that RVVC should not necessarily be linked to sexual activity.

This present study estimated feminine/vaginal wash use among women in the Sekondi/Takoradi metropolis and reported a prevalence of 47.7%. This indicates that feminine/vaginal wash use is widespread in the Sekondi-Takoradi metropolis. Our estimate, however, is slightly lower than the estimates of 67.7% reported by Ziba et al., (2019) among Ghanaian women residing in Bolgatanga [13] and 79.0% reported by Hou et al. among adolescents admitted to a correctional institution for girls in the USA [26]. In Thailand, Mozambique and South Africa, the prevalence of feminine/vaginal wash use was estimated to be above 70%, 60% and 90% respectively [27, 28]. All these estimates are higher than the estimate from Sekondi-Takoradi which is being reported by this present study.

The use of feminine wash is prevalent globally and not limited to a particular geographical location, ethnicity, or culture. The respondents’ use of feminine/vaginal washes was distributed in this study, with no statistically significant association determined within the sociodemographic variables.

The etiology of RVVC is multifactorial, involving both endogenous and exogenous factors [19]. One exogenous factor that garnered attention is the use of feminine wash products. This study investigated the potential association between feminine wash use and the development of recurrent vulvovaginal candidiasis.

Our study found a statistically significant association between using feminine/vaginal washes and developing RVVC, as the odds of developing RVVC were almost 4 times higher in women who used vaginal washes than in women who did not use feminine/vaginal washes. This finding specifically agrees with a study performed in China that reported higher odds of RVVC among women who used vaginal washes [29]. Apart from RVVC, many studies have reported higher odds of developing Urinary Tract Infections [UTIs), Sexually Transmitted Infections (STIs) and HIV among women who use feminine hygiene products [30, 31]. The safety and efficacy of feminine/vaginal wash products have been contested by several researchers, with some researchers cautioning against their use because they could be harmful [4, 32]. The vaginal microflora is delicate, and a disturbance that alters this delicate balance can lead to persistent infection, such as RVVC [33]. Using these products over time can alter the vaginal flora and allow for persistent opportunistic yeast infections [24]. Therefore, the use of these products should be cautioned, and women need to be educated on the possible dangers these products may have on their health.

### Study limitations

Several factors must be considered when interpreting these findings. First, the study’s design plays a crucial role in determining the reliability of the results. This study employed a cross-sectional study design, so the observed association may not necessarily imply a causal relationship. Variables such as personal hygiene, use of public washrooms, sexual activity and endogenous variables such as the virulence of the strain of candida and the innate immunity of the participants were not evaluated. Such variables can distort the true nature of the association, resulting in a spurious relationship.

Moreover, recall bias and social desirability bias could affect the accuracy of the reported feminine wash use. Additionally, the study needed to account for the specific ingredients in various feminine wash products, as numerous formulations on the market have different ingredients. Different formulations may have varying impacts on the vaginal microbiota and susceptibility to RVVC. To establish a more conclusive understanding, prospective studies that consider diverse formulations of feminine washes, accurate measurements and control for confounders are essential to establish causality.

## Conclusion

This study estimated a high prevalence of RVVC among women of reproductive age in the Secondi/Takoradi Metropolis. The study found age, marital status, and level of sexual activity to be significantly associated with RVVC. Most importantly a statistically significant association emerged between the use of feminine/vaginal washes use and the development of recurrent VVC. This raises important questions about the potential impact of hygiene practices on vaginal health of women. To establish a more conclusive understanding, prospective studies that consider diverse formulations of feminine washes, accurate measurements and control for confounders are essential to establish causality.

## Data Availability

The data used in this study are available upon reasonable request from the corresponding author.

## Abbreviations

AIDS: Acquired Immune Deficiency Syndrome
CDC: Centers for Disease Control and Prevention
HIV: Human Immunodeficiency Virus
RVVC: Recurrent Vulvovaginal Candidiasis
VVC: Vulvovaginal Candidiasis
